# Community-Engaged Research: COVID-19 Testing, Infection, and Vaccination among Underserved Minority Communities in Miami, Florida

**DOI:** 10.3390/vaccines12020117

**Published:** 2024-01-24

**Authors:** Haley R. Martin, David R. Brown, Eileen Fluney, Mary Jo Trepka, Aileen M. Marty, Eneida O. Roldan, Qingyun Liu, Manuel A. Barbieri, Marianna K. Baum

**Affiliations:** 1 Department of Dietetics and Nutrition, Robert Stempel College of Public Health and Social Work, Florida International University, 11200 SW 8th Street, AHC5, Miami, FL 33199, USA; halmarti@fiu.edu (H.R.M.); qliu@fiu.edu (Q.L.); 2 Herbert Wertheim College of Medicine, Florida International University, 11200 SW 8th Street, AHC2, Miami, FL 33199, USA; drbrown@fiu.edu (D.R.B.); amarty@fiu.edu (A.M.M.); eoroldan@fiu.edu (E.O.R.); 3 Paradise Christian School, 6184 W 21st Court, Hialeah, FL 33016, USA; dreileenfluney@gmail.com; 4 Department of Epidemiology, Robert Stempel College of Public Health and Social Work, Florida International University, 11200 SW 8th Street, AHC5, Miami, FL 33199, USA; trepkam@fiu.edu; 5 Department of Biological Sciences, College of Arts, Sciences & Education, Florida International University, 11200 SW 8th Street, OE 167, Miami, FL 33199, USA; barbieri@fiu.edu

**Keywords:** underserved populations, community-engaged research, COVID-19, testing, infection, vaccination

## Abstract

Community collaboration is a cornerstone of modern public health efforts. This work aimed to use community-engaged research to explore COVID-19 vaccination, testing, and infection in a minoritized community. This study was conducted in Miami, Florida, from March 2021 to February 2022 in community partner sites and the Miami Adult Studies on HIV (MASH) cohort. Sociodemographic characteristics, vaccination and testing beliefs, and COVID-19 challenges were self-reported. COVID-19 vaccinations were verified with medical records, testing history was self-reported, and severe acute respiratory syndrome coronavirus 2 positivity was determined via real-time reverse transcription–polymerase chain reaction (rt-PCR). Of 1689 participants, the median age was 57, 51% were male, 49% were non-Hispanic Black, 66% reported an income < USD 15,000/year, and 75.9% received at least one dose of a COVID-19 vaccine. Belief that COVID-19 vaccination is effective was associated with lower odds of COVID-19 positivity and was the strongest predictor of vaccination. Challenges accessing health care, housing, food, and transportation were associated with lower odds of vaccination. Employment, health insurance, higher education, and greater perceived test accuracy were associated with greater odds of COVID-19 testing. Social determinants of health and the belief that vaccines are effective and tests are accurate predicted behaviors and thus should be considered during public health crises in vulnerable communities.

## 1. Introduction

Community involvement and collaboration are cornerstones of modern public health improvement efforts [1]. Community-engaged research incorporates meaningful participation from those whom the research outcomes will reasonably impact [1,2]. Community-engaged research is a valuable way to solve some of the most pressing and complex health problems by maintaining meaningful partnerships with community-based organizations, mobilizing resources, and building trust [2], and was an effective tool during the COVID-19 pandemic [3,4]. Public health crises such as the COVID-19 pandemic often amplify economic and health disparities, disproportionately affecting underserved communities [5]. For example, severe COVID-19 disease has been associated with lower socioeconomic status (SES), densely populated communities, homelessness, food insecurity, and minoritized groups [6].

Vaccination and testing are essential to contagion containment. Yet, disparities in vaccination, testing, and infection by SES, thought to be related to social determinants of health and health beliefs, are of concern in underserved and minoritized communities. Although typically not uniform across the United States (U.S.), disparities in COVID-19 testing rates by race and ethnicity have been reported [7], and those with lower SES (lower income, education, and health insurance coverage) have less access to COVID-19 testing [8] and lower vaccination rates [9], which has been linked to higher rates of COVID-19 in these minoritized populations [10]. Altogether, this indicates the potential impact of social determinants of health and health beliefs on testing and vaccination behaviors, which may ultimately contribute to disparities in infection and disease severity. 

Miami, Florida experiences a high level of social vulnerability; the COVID-19 Community Vulnerability Index scored Miami-Dade County as 0.90, which indicates “very high” COVID-19 vulnerability [11]. Indeed, we have found our project participants to be a socially vulnerable population who experience high rates of poverty, housing instability, food insecurity, disability, and substance abuse [12,13,14]. In this study, we employed community-engaged research to explore if social determinants of health and health beliefs could predict COVID-19 vaccination, testing, and infection among an underserved, minoritized community in Miami with the National Institutes of Health (NIH) Rapid Acceleration of Diagnostics—Underserved Populations (RADx-UP) initiative.

## 2. Materials and Methods

### 2.1. Study Design and Participants

The NIH RADx-UP initiative is a consortium of over 135 projects studying COVID-19 testing patterns using a community-engaged approach in historically minoritized communities across the U.S. with the aim of speeding up innovation in the implementation of severe acute respiratory syndrome coronavirus 2 (SARS-CoV-2) testing [15]. RADx-UP projects collect a set of common data elements (CDEs) (available at https://radx-up.org/research/cdes/) (accessed on 10 December 2023) that are then uploaded to the RADx-UP Coordination and Data Collection Center (CDCC) led by the Duke Clinical Research Institute at Duke University (Durham, North Carolina) and the Center for Health Equity Research at the University of North Carolina at Chapel Hill. The RADx-UP CDEs include items from the NIH CDE Repository, Disaster Research Response (DR2) guidelines, and the PhenX Toolkit. The RADx-UP consortium selected, developed, and refined community-informed CDEs via an iterative process to support standardized data collection and promote cross-consortium data harmonization and analysis [16]. 

The data reported were from an individual RADx-UP project located in Miami, Florida; data were collected between March 2021 and February 2022. This specific RADx-UP project aimed to provide COVID-19 testing to underserved communities in Miami while also exploring the ability of these communities to access and utilize COVID-19 testing, vaccination, and treatment. We collaborated with community partners to recruit participants and provide testing at our community clinic and via community testing events with community-based organization partner sites such as churches and schools in socioeconomically disadvantaged neighborhoods. Recruitment was also carried out at food banks and within the Miami Adult Studies on HIV (MASH) cohort, an NIH-funded cohort that follows over 1000 underserved, mostly Black and Hispanic adults, living with and without HIV and other comorbidities. This RADx-UP project also employed a Scientific Advisory Board and Community Advisory Board to guide our protocol, encourage participant enrollment, and help reach underrepresented populations [17]. We administered questionnaires by phone. In-person interviews were conducted for participants with lack of access to a working phone or with communication difficulties. This RADx-UP project included adults aged 18 years or older, while those who were pregnant or unwilling to complete the questionnaire or testing were excluded. Eligible participants subsequently completed the questionnaire with trained research staff, which included RADx-UP CDEs plus additional items implemented by our specific project site. After completing the questionnaire, participants were provided an appointment for real-time reverse transcription–polymerase chain reaction (rt-PCR) COVID-19 testing at our community clinic or at our community testing events at community-based organization partner sites. This specific analysis included participants who completed both the questionnaire and rt-PCR COVID-19 testing at a Clinical Laboratory Improvement Amendments (CLIA)-certified laboratory. The Florida International University Institutional Review Board approved the study protocol (IRB-15-0004) and all participants provided informed consent. Additionally, the RADx-UP CDCC was approved by the IRB at Duke University, and all participants provided informed consent to share data.

### 2.2. Outcomes

The primary outcomes of this analysis were the odds of COVID-19 vaccination, testing, and infection. COVID-19 vaccination history was self-reported by participants and confirmed with medical records when available; 82.2% of vaccinations were confirmed through medical records. History of COVID-19 testing was assessed with modified items from PhenX Toolkit protocols [18]. COVID-19 infection history was self-reported by the participants. COVID-19 positivity during this study was assessed with rt-PCR testing. Samples were collected at our clinic located within the minoritized communities we collaborate with and also at community testing events, which were led by community partners in additional underserved areas of Miami, Florida.

### 2.3. Predictors and Covariates

Elements of social determinants of health such as income, employment, health care and insurance, education, transportation, housing, and food access, along with race/ethnicity and health beliefs regarding testing and vaccination, were the main focus of the chosen predictors of interest. We collected sociodemographic characteristics by utilizing PhenX protocols that were modified and refined by RADx-UP [19]. Housing insecurity was assessed with the Veterans Affairs Homelessness Screening Clinical Reminder [20], and the University of Michigan Detroit Metro Area Communities Study (DMACS) COVID-19 Survey was used to assess health insurance status [21]. We assessed COVID-19-related challenges by utilizing items adapted for the Community Engagement Alliance Against COVID-19 from the DMACS COVID-19 Survey and included difficulties accessing health care, housing, food, drinking water, medications, and transportation [21]. Additional challenges included loss of health care coverage due to the COVID-19 pandemic. Greater perceived accuracy of a positive/negative COVID-19 test result was defined as a response of “Somewhat confident”, “Confident”, or “Very confident” to “How confident are you that a positive test result means that you do have COVID-19?” and “How confident are you that a negative test result means that you do not have COVID-19?” High COVID-19 worry was defined as a response of ≥6 to “On a scale of 1 to 10, how worried are you about the COVID-19 pandemic?” The belief that COVID-19 vaccination is effective was defined as a response of “Agree” to “COVID-19 vaccination is an effective way to prevent and control COVID-19”. Chronic health conditions were determined using the Johns Hopkins University C4-Ward Module Five: Comorbidities and Care Engagement [22]. We confirmed HIV serostatus with medical records while antiretroviral therapy use was self-reported. HIV viral load and CD4 cell count were abstracted from medical records for those living with HIV, following written consent.

### 2.4. Statistical Analyses

The initial sample size for this RADx-UP project was determined with the primary goal of testing more participants per week than a leading community health center in Miami; our project goal was to test 1600 adults from vulnerable communities. We reported descriptive statistics as *n* (%) and mean ± standard deviation (SD) or median and interquartile range (IQR) for categorical and continuous variables, respectively. Normality of the distribution of continuous variables was assessed with the Shapiro–Wilk test and examination of histograms. Differences between groups were evaluated with the chi-square test for categorical outcomes (Fisher’s exact test was employed for small cell counts) and *t*-tests for continuous outcomes (the Wilcoxon rank-sum test was utilized for skewed distributions). We employed logistic regression to estimate odds ratios (ORs) with corresponding 95% confidence intervals (CIs). All multivariable models were adjusted for age, sex, race/ethnicity, and income. For the models with COVID-19 infection as the outcome, morbidity burden and COVID-19 vaccination were also included as covariates. For models with race/ethnicity as the main predictor, race/ethnicity was not included as a covariate. Similarly, for models with income as the main predictor, income was not included as a covariate. For models with belief that COVID-19 vaccination is effective as the main predictor and COVID-19 infection as the outcome, COVID-19 vaccination was not included as a covariate to prevent collinearity. We calculated variance inflation factors to ensure the absence of collinearity in all models. Missing data were not dependent on the main outcomes of interest and were, therefore, considered missing at random and excluded [23]. Statistical significance was set at two-tailed *p* < 0.05 and all analyses were conducted using SAS OnDemand for Academics, Version 9.4 (SAS, Inc., Cary, NC, USA).

## 3. Results

### 3.1. Sample Characteristics

A total of 1785 participants consented to participate and were enrolled, 1781 completed the screening, and 1755 completed the questionnaire (26 participants were ineligible or did not complete the questionnaire for some other reason). Among the 1755 participants who completed the questionnaire, 66 (3.8%) did not attend their testing appointment; no differences were observed between participants who had ever received a COVID-19 test prior to participating in this study and those who had never received a test prior to participating (3.9% vs. 3.2%, *p* = 0.559). The final sample size of this analysis was 1689; 31.0% were MASH cohort participants. Thus, we surpassed our initial project goal of testing 1600 adults from underserved communities. A total of 276 participants (16.3%) had never received a COVID-19 test prior to participating in this study (Table 1). Compared to those who had ever received a COVID-19 test, those who had never been tested before were more likely to identify as Black non-Hispanic, have less than high school education, have a disability that impacted employment status, have a household income of less than USD 15,000/year, and be living with HIV (Table 1). 

### 3.2. Predictors of COVID-19 Vaccination

A total of 1281 participants (75.9%) received at least one dose of a COVID-19 vaccine. Multivariable analyses were adjusted for age, sex, race/ethnicity, and income. Challenges with obtaining necessary health care, housing insecurity, insufficient food, and transportation challenges were all associated with lower odds of COVID-19 vaccination. Compared to White non-Hispanic participants, those who identified as Black non-Hispanic had the lowest odds of COVID-19 vaccination. Loss of health insurance coverage due to the COVID-19 pandemic was also associated with lower odds of vaccination. Conversely, health insurance coverage, higher education level, high COVID-19-related worry, and the belief that COVID-19 vaccination is effective were associated with greater odds of vaccination (Table 2).

### 3.3. Predictors of Ever Being Tested for COVID-19

We conducted multivariable analyses adjusted for age, sex, race/ethnicity, and income. Compared to White non-Hispanic participants, those who identified as Black non-Hispanic were less likely to have received a COVID-19 test. Current employment, health insurance coverage, higher education level, greater perceived accuracy of a positive COVID-19 test result, high COVID-19-related worry, and receipt of a COVID-19 vaccine were all associated with greater odds of COVID-19 testing (Table 3).

### 3.4. Predictors of COVID-19 Infection

A total of 264 participants (15.6%) self-reported a past COVID-19 infection. To assess the relationship between potential predictors and odds of a past COVID-19 infection, multivariable logistic regressions adjusted for age, sex, race/ethnicity, income, morbidity burden, and vaccination status were performed (Table 4). Race/ethnicity was a significant predictor. Compared to White non-Hispanic participants, those who identified as Black Hispanic or White Hispanic had greater odds of a past COVID-19 infection. Higher income and greater perceived accuracy of a positive COVID-19 test result were also associated with greater odds of a past COVID-19 infection (Table 4).

Under 3% of participants (2.8%, *n* = 47) tested positive for COVID-19 at the time of the study via rt-PCR testing at our community testing sites. We conducted separate multivariable logistic regressions adjusted for age, sex, race/ethnicity, income, morbidity burden, and vaccination status (Table 5). The belief that COVID-19 vaccination is effective was the only predictor associated with lower odds of COVID-19 positivity via rt-PCR testing (Table 5).

## 4. Discussion

We present findings from a community-engaged research project within the NIH RADx-UP initiative in which we recruited, with the help of community partners, a large sample of adults from racial and ethnic minority backgrounds with low employment, education, and income in underserved areas of Miami, Florida. In this study, we aimed to provide COVID-19 testing to underserved communities in Miami while also exploring potential predictors of COVID-19 vaccination, testing, and infection. We successfully provided testing to more than 1600 participants at our community clinic and via community testing events with community-based organization partner sites such as churches and schools in socioeconomically disadvantaged neighborhoods; an important contribution to the community being that Miami, Florida experiences a high level of social and COVID-19 vulnerability according to the COVID-19 Community Vulnerability Index [11]. Importantly, we found several significant predictors of COVID-19 vaccination, testing, and infection among a minoritized community. The overall findings indicate that race and ethnicity, social determinants of health, and the belief that vaccines are effective and tests are accurate predicted behaviors.

Compared to those who had received a COVID-19 test, those who had never been tested before were more likely to identify as Black non-Hispanic, have less education and lower income, and were less likely to be employed. We found that, compared to White non-Hispanic participants, those who identified as Black non-Hispanic were less likely to have received a COVID-19 test and had the lowest odds of COVID-19 vaccination. These findings of disparities in COVID-19 testing and vaccination rates by race and ethnicity concur with other published findings [7,9]. Additionally, results from Drefahl et al. demonstrate that in Sweden, those with lower income and education were also more likely to die from severe COVID-19, suggesting disparities in income and education may contribute to even worse COVID-19 outcomes internationally [24]. We provide evidence that these disparities existed a year later into the pandemic (March 2021–February 2022) in Miami, and that Black adults may have considerably higher rates of undiagnosed COVID-19 infection.

Current employment, health insurance coverage, and higher education were associated with greater odds of COVID-19 testing. Despite COVID-19 testing being provided at no charge at the time these data were collected, our findings agree with Ali et al., who reported higher income and health insurance coverage to be associated with perceived access to COVID-19 testing [8]. This suggests that campaigns advertising testing as free, regardless of health insurance status, were not reaching or trusted by certain communities and indicates a need for stronger and better tailored population-wide communication during future public health crises [8]. This finding could also indicate that those with health insurance were simply more engaged in health care behaviors and habits both prior to and during the COVID-19 pandemic, indicating the importance of health insurance coverage in health care access and engagement in preventive health behaviors. It is also possible that uninsured health status may impact preventive health behaviors due to disengagement from the health care system across the lifespan. Apart from actual access to testing, several factors may play a role in the decision to be tested for COVID-19, such as perceived need to be tested, perceived safety of the test, and perceived risk of severe COVID-19 illness [8]. We found that greater perceived accuracy of a positive COVID-19 test result and high COVID-19-related worry were associated with greater odds of COVID-19 testing. This adds to the growing literature in support of health beliefs and attitudes predicting preventive behaviors [25], but extends it to predicting COVID-19 testing specifically in a minoritized community.

Similarly, we found that the belief that COVID-19 vaccination is effective was the strongest predictor of COVID-19 vaccination; those who believed the vaccine is effective had over 12 times the odds of being vaccinated than those who did not believe it was effective. High COVID-19-related worry was also associated with greater odds of vaccination. These findings reinforce the Health Belief Model framework that focuses on individual attitudes and beliefs and includes the following constructs to predict the likelihood of a health behavior: perceived susceptibility to illness, perceived severity of illness, perceived benefits of preventative behavior, perceived barriers, cues to action, and self-efficacy [25]. The underlying concept of the Health Belief Model is that health behaviors may be determined by personal beliefs about a disease and the strategies available to prevent it [25]. Indeed, a study conducted in Romania reported that worry about the COVID-19 pandemic predicted vaccination directly and indirectly through the perceived threat of disease and benefits of vaccination [26]. We found that the belief that tests are accurate and vaccines are effective predicted COVID-19 preventive health behaviors in a minoritized community in the U.S., thus adding to the literature supporting the role of personal beliefs and attitudes in preventive behaviors globally.

When we consider the time our data were collected (March 2021 to February 2022), according to the U.S. Department of Health and Human Services’ COVID-19 vaccination schedule, as of April 2021, all people in the U.S. aged 16 and older were eligible for the COVID-19 vaccine (primary two-dose or one-dose series, depending on the manufacturer of the vaccine). This was followed by a directive to allow a booster dose to certain eligible populations in September 2021 and a booster dose to all adults 18 years and older in November 2021 [27]. We observed high vaccination rates in our sample; over 75% had received at least one dose of a COVID-19 vaccine. However, despite the effectiveness of COVID-19 vaccination in preventing severe illness, hospitalization, and death due to COVID-19 [28], vaccination rates remain low among minoritized populations in the U.S. [29]. Barriers to vaccination include poor access to vaccination sites, fear of mistreatment by medical professionals, mistrust of the medical community, lack of transportation, limited health care access, concerns about stigma and discrimination, and complexity of booking systems [30]. We found that challenges with obtaining necessary health care, housing insecurity, insufficient food, and transportation challenges were all associated with lower odds of COVID-19 vaccination. Challenges obtaining health care and transportation may stand as direct barriers to accessing vaccination sites, and challenges with housing and food insecurity may lead to competing priorities. Loss of health insurance coverage due to the COVID-19 pandemic was also associated with lower odds of vaccination. Conversely, health insurance coverage and higher education were associated with greater odds of vaccination. These findings agree with previous literature that reported positive associations between SES (higher income, more education, and higher employment) and health insurance coverage with COVID-19 vaccination [9]. However, our findings extend this literature by highlighting the potential role that additional social determinants of health such as housing, food insecurity, and transportation also play in COVID-19 vaccination. We also demonstrate the meaningful role that community-engaged research may play in exploring COVID-19 vaccination uptake.

Our results demonstrate the impact of social determinants of health on COVID-19 disparities, with employment, education, and health insurance predicting COVID-19 testing and vaccination. Social determinants of health are defined as the conditions in the environment that affect health, functioning, and quality of life and include five major domains: economic stability, education access and quality, health care access and quality, neighborhood and built environment, and social and community context [31]. Simply making COVID-19 testing and vaccination available and promoting them will not eliminate these disparities [31]. Instead, public health efforts should employ community-engaged research to improve the social, physical, and economic conditions in the environments of underserved communities to improve employment opportunities, education, and health insurance accessibility and utilization [31].

At the time these data were collected (March 2021–February 2022), COVID-19 testing and vaccines were available for free in the U.S., regardless of insurance status. However, the end of the Federal COVID-19 Public Health Emergency Declaration in May 2023 marked the beginning of changes to the availability of COVID-19 testing and vaccination [32]. For example, insurance providers are no longer required to waive costs or provide free COVID-19 tests [32]. We present evidence of disparities in COVID-19 testing and vaccination when these services were provided for free, and therefore, we anticipate these disparities to widen now that testing and vaccination may impose financial burdens on uninsured, low-income people.

Over 15% of participants self-reported a past COVID-19 infection, and under 3% tested positive for COVID-19 at the time of this study via rt-PCR testing. Compared to White non-Hispanic participants, those who identified as Black or Hispanic presented with greater odds of a past COVID-19 infection. Others have confirmed this finding [7,10]. Jacobson et al., for example, reported that Hispanic adults in California presented with greater odds of a positive COVID-19 test, as well as greater odds of COVID-19 hospitalization and death earlier in the pandemic (February 2020–March 2021) compared to White non-Hispanic adults [7]. Another study conducted in Brazil by Baqui et al. similarly reported worse COVID-19 outcomes in mixed-ethnicity and Black Brazilians, compared with White Brazilians [33], demonstrating that racial/ethnic health disparities in COVID-19 outcomes are a global issue. This research provides evidence of additional racial/ethnic disparities in COVID-19 infection one year into the pandemic (March 2021–February 2022) in the Southeastern U.S. Importantly, we found that the belief that COVID-19 vaccination is effective was associated with significantly lower odds of COVID-19 positivity via rt-PCR testing. This finding supports the Health Belief Model, as beliefs about vaccination efficacy predicted the decision to receive the COVID-19 vaccine, and were also associated with lower odds of COVID-19 positivity.

Strengths of the present study include our community-engaged approach and employment of community partnerships to successfully engage and provide testing to a large sample of underserved, minoritized adults. This research demonstrates the impact that community-engaged research can have in exploring predictors of COVID-19 vaccination, testing, and infection. Challenges of the presented research included timely notification of COVID-19 test results in “hard-to-reach” populations. However, our community partnerships remained central to engagement efforts. Through these partnerships, we were able to develop a successful and appropriate protocol, encourage participant enrollment, and engage a large sample of minoritized adults. This study is limited by its cross-sectional design and inclusion of some self-reported data, which are susceptible to recall bias and underreporting. We also did not consider COVID-19 disease severity, which is important to consider as it is a contributor to COVID-19 hospitalization and death [7]. Additionally, re-infection or multiple infections of SARS-CoV-2 could not be assessed. Beyond that, due to the multiple category levels in the race/ethnicity variable, there were multiple comparisons made within that variable. However, given that this analysis was exploratory in nature, multiple-comparison corrections were not made and thus findings regarding race/ethnicity as a predictor should be interpreted with caution. Lastly, the results of the present study are not intended to be generalizable to the general U.S. population or the general population in Miami, as we did not employ random sampling. Our intent was to recruit minoritized adults.

## 5. Conclusions

This community-engaged research successfully provided COVID-19 testing to more than 1600 vulnerable adults at our community clinic and via community testing events with community-based organization partner sites. We found that greater education and higher rates of employment and health insurance coverage were associated with greater odds of COVID-19 vaccination and testing, demonstrating the impact of social determinants of health on COVID-19 disparities. Additionally, the belief that COVID-19 tests are accurate and COVID-19 vaccines are effective predicted testing and vaccination behaviors and was associated with lower odds of COVID-19 infection, reinforcing the Health Belief Model framework that contends health behaviors and subsequent outcomes are at least partially determined by personal beliefs and perceptions. These findings extend the literature by highlighting the potential role that additional social determinants of health such as challenges with housing, food insecurity, and transportation play in COVID-19 vaccination. We also demonstrated the meaningful role that community-engaged research may play in providing testing and exploring vaccination uptake in underserved communities that are most vulnerable to public health crises. Public health efforts should consider meaningful participation from minoritized communities through the use of community-engaged research to improve the social, physical, and economic conditions in underserved communities and thereby improve employment opportunities, education, and health insurance accessibility and utilization. Community-engaged health care delivery should be considered to help mitigate disparities in testing and vaccination during public health crises. Beliefs about test accuracy and vaccine efficacy should be considered in messaging interventions aimed at increasing testing and vaccine uptake in underserved communities. Transparency of research and trust in the messengers who promote health behaviors, which are pillars of community-engaged research, are essential for the success of interventions aimed at health beliefs in minoritized populations who have historically been disenfranchised from access to health care.

## Figures and Tables

**Table 1:** Characteristics of participants by COVID-19 testing history.

	Total	Received ≥ 1 COVID-19 Test	Never Been Tested for COVID-19	
**Variable ^a^**	**(*n* = 1689)**	**(*n* = 1413, 83.7%)**	**(*n* = 276, 16.3%)**	***p***
Age, years, median (IQR)	57.0 (49.0–63.0)	57.0 (48.0–63.0)	58.0 (51.0–64.0)	0.101
Male sex assigned at birth	854 (50.6)	705 (49.9)	149 (54.0)	0.214
Race/Ethnicity (*n* = 1682) ^b^				
Black non-Hispanic	816 (48.5)	641 (45.6)	175 (63.6)	<0.001 *
Black Hispanic	73 (4.3)	64 (4.6)	9 (3.3)	
White non-Hispanic	92 (5.5)	82 (5.8)	10 (3.6)	
White Hispanic	610 (36.3)	539 (38.3)	71 (25.8)	
Other/Multiracial ^c^	91 (5.4)	81 (5.8)	10 (3.6)	
Education (*n* = 1686) ^b^				
Less than high school	571 (33.9)	453 (32.1)	118 (42.9)	<0.001 *
High school or GED	492 (29.2)	395 (28.0)	97 (35.3)	
Some college or more	623 (37.0)	563 (39.9)	60 (21.8)	
Household				
Number in household (*n* = 1686), median (IQR) ^b^	2.0 (1.0–3.0)	2.0 (1.0–3.0)	2.0 (1.0–2.5)	0.312
Lives alone	675 (40.0)	564 (39.9)	111 (40.2)	0.003 *
Lives with spouse	300 (17.8)	232 (16.4)	68 (24.6)	
Lives in multi-generational home	435 (25.8)	371 (26.3)	64 (23.2)	
Lives in other type of household	279 (16.5)	246 (17.4)	33 (12.0)	
Employment (*n* = 1683) ^b^				
Working now	369 (21.9)	336 (23.9)	33 (12.0)	<0.001 *
Unemployed	374 (22.2)	300 (21.3)	74 (27.0)	
Retired	219 (13.0)	181 (12.9)	38 (13.9)	
Disabled	625 (37.1)	507 (36.0)	118 (43.1)	
Other	96 (5.7)	85 (6.0)	11 (4.0)	
Experienced loss of employment income during the pandemic	537 (31.8)	473 (33.5)	64 (23.2)	<0.001 *
Health insurance (*n* = 1677) ^b^				
None/Do not know	315 (18.8)	254 (18.1)	61 (22.3)	<0.001 *
Private	254 (15.2)	238 (17.0)	16 (5.8)	
Public (Medicare, Medicaid, Tricare)	1108 (66.1)	911 (64.9)	197 (71.9)	
Income (*n* = 1688) ^b^				
Less than USD 15,000	1110 (65.8)	909 (64.4)	201 (72.8)	0.006 *
USD 15,000–USD 34,999	382 (22.6)	328 (23.2)	54 (19.6)	
USD 35,000–USD 74,999	102 (6.0)	96 (6.8)	6 (2.2)	
USD 75,000 and more	20 (1.2)	19 (1.4)	1 (0.4)	
Prefer not to answer	74 (4.4)	60 (4.3)	14 (5.1)	
Chronic health conditions (*n* = 1685) ^b,d^				
0	310 (18.4)	253 (17.9)	57 (20.7)	0.706
1	365 (21.7)	306 (21.7)	59 (21.5)	
2–3	569 (33.8)	477 (33.8)	92 (33.5)	
4+	441 (26.2)	374 (26.5)	67 (24.4)	
Fair/Poor self-reported health (*n* = 1686) ^b^	339 (20.1)	293 (20.8)	46 (16.7)	0.126
COVID-19 vaccination, ≥1 dose (*n* = 1688) ^b^	1281 (75.9)	1115 (79.0)	166 (60.1)	<0.001 *
Living with HIV	411 (24.3)	318 (22.5)	93 (33.7)	<0.001 *
On antiretroviral therapy (*n* = 411)	399 (97.1)	312 (98.1)	87 (93.6)	0.913
Virally suppressed (*n* = 231) ^b,e^	192 (83.1)	147 (84.0)	45 (80.4)	0.527
CD4 lymphocyte count, cells/µL (*n* = 242), median (IQR) ^b,e^	561.5 (383.0–851.0)	572.0 (397.0–875.0)	558.0 (356.0–718.0)	0.226

Note. GED, General Educational Development; IRQ, interquartile range. ^a^ Data are presented as *n* (%) for categorical variables and median (IQR) for continuous variables. ^b^ Missing data. ^c^ “Other race” included Asian, Native Hawaiian or Other Pacific Islander, American Indian or Alaska Native, some other reported race that was not included in the response options, or report of multiple (more than one) races. ^d^ Chronic conditions included immunocompromising conditions, autoimmune diseases, hypertension, diabetes, kidney diseases, cancer, cardiovascular disease, asthma, chronic obstructive pulmonary disease, other lung diseases, sickle cell anemia, depression, substance use disorders, obesity, and other mental health disorders. ^e^ Viral suppression was defined as a viral load of <200 copies of HIV/mL. HIV viral load and CD4 lymphocyte data were abstracted from medical records; values within 4 months of data collection were considered. * *p* < 0.05.

**Table 2:** Predictors of receiving ≥ 1 dose of a COVID-19 vaccine: Univariate and multivariable binary logistic regressions ^a^.

Predictor	OR	95% CI	*p*	aOR ^b^	95% CI	*p*
COVID-19-related challenges; challenges with:						
Obtaining necessary health care (including for mental health)	0.63	0.49–0.82	0.001 *	0.71	0.54–0.93	0.014 *
Having a place to stay/live	0.57	0.43–0.75	<0.001 *	0.66	0.49–0.88	0.005 *
Sourcing enough food to eat	0.60	0.47–0.77	<0.001 *	0.68	0.52–0.88	0.004 *
Having clean water to drink	0.67	0.47–0.97	0.034 *	0.74	0.50–1.08	0.117
Obtaining necessary medicine	0.73	0.53–0.99	0.042 *	0.83	0.60–1.14	0.248
Transportation	0.59	0.46–0.77	<0.001 *	0.68	0.52–0.90	0.008 *
Race/Ethnicity (*n* = 1682)						
Black non-Hispanic vs. White non-Hispanic	0.48	0.28–0.83	0.008 *	0.46	0.26–0.81	0.007 *
Black Hispanic vs. White non-Hispanic	0.64	0.31–1.35	0.245	0.69	0.32–1.49	0.345
White Hispanic vs. White non-Hispanic	1.26	0.71–2.23	0.431	1.36	0.76–2.45	0.299
Other/Multiracial ^c^ vs. White non-Hispanic	1.06	0.50–2.26	0.875	1.29	0.59–2.80	0.524
Higher income (*n* = 1688) ^d^	1.22	0.95–1.57	0.120	1.24	0.94–1.63	0.122
Current employment (*n* = 1683)	1.10	0.84–1.45	0.487	1.21	0.88–1.68	0.247
Current health insurance coverage (*n* = 1677) ^e^	2.20	1.69–2.86	<0.001 *	2.12	1.59–2.81	<0.001 *
Loss of health insurance coverage due to the COVID-19 pandemic (*n* = 1687)	0.60	0.39–0.92	0.020 *	0.56	0.36–0.89	0.014 *
Higher education level (*n* = 1686) ^f^	1.61	1.22–2.11	0.001 *	1.46	1.08–1.96	0.013 *
Greater perceived accuracy of positive COVID-19 test result (*n* = 1688)	1.62	1.21–2.17	0.001 *	1.33	0.97–1.81	0.076
Greater perceived accuracy of negative COVID-19 test result	1.29	0.75–2.22	0.366	1.03	0.58–1.81	0.931
High COVID-19-related worry	1.53	1.22–1.92	<0.001 *	1.43	1.12–1.83	0.004 *
Belief that COVID-19 vaccination is effective	13.95	9.10–21.39	<0.001 *	12.75	8.11–20.05	<0.001 *

Note. aOR, adjusted odds ratio; OR, odds ratio; 95% CI, 95% confidence interval. ^a^ According to the United States (U.S.) Department of Health and Human Services, the COVID-19 vaccination schedule in the U.S. during the study period (March 2021 to February 2022) indicated that as of April 2021, all people in the U.S. aged 16 and older were eligible for the COVID-19 vaccine (primary two-dose or one-dose series, depending on the manufacturer of the vaccine), followed by a directive to allow a booster dose to certain eligible populations in September 2021 and a booster dose to all adults 18 years and older in November 2021. ^b^ Multivariable models adjusted for age, sex, race/ethnicity, and income. Race/ethnicity was not included as a covariate in the model with race/ethnicity as the main predictor. Income was not included as a covariate for the model with higher income as the main predictor. ^c^ ”Other race” included Asian, Native Hawaiian or Other Pacific Islander, American Indian or Alaska Native, some other reported race that was not included in the response options, or report of multiple (more than one) races. ^d^ Annual household income ≥USD 15,000 vs. <USD 15,000. “Prefer not to answer” responses were separated into a third category. ^e^ Private or public insurance (Medicare, Medicaid, Tricare) vs. no insurance/do not know. ^f^ Some college or more vs. less than high school. * *p* < 0.05.

**Table 3:** Predictors of ever being tested for COVID-19: Univariate and multivariable binary logistic regressions.

Predictor	OR	95% CI	*p*	aOR ^a^	95% CI	*p*
COVID-19-related challenges; challenges with:						
Obtaining necessary health care (including for mental health)	0.89	0.65–1.22	0.468	0.90	0.65–1.24	0.508
Having a place to stay/live	1.34	0.93–1.94	0.112	1.41	0.97–2.05	0.073
Sourcing enough food to eat	0.88	0.65–1.18	0.384	0.93	0.68–1.25	0.611
Having clean water to drink	0.90	0.58–1.41	0.644	0.95	0.60–1.49	0.809
Obtaining necessary medicine	1.00	0.69–1.45	0.986	1.00	0.68–1.46	0.991
Transportation	1.03	0.74–1.43	0.875	1.08	0.77–1.51	0.674
Race/Ethnicity (*n* = 1682)						
Black non-Hispanic vs. White non-Hispanic	0.45	0.23–0.88	0.020 *	0.47	0.24–0.93	0.029 *
Black Hispanic vs. White non-Hispanic	0.87	0.33–2.26	0.771	0.89	0.34–2.32	0.808
White Hispanic vs. White non-Hispanic	0.93	0.46–1.87	0.830	0.92	0.45–1.85	0.804
Other/Multiracial ^b^ vs. White non-Hispanic	0.99	0.39–2.50	0.979	0.98	0.39–2.48	0.962
Higher income (*n* = 1688) ^c^	1.61	1.18–2.19	0.003 *	1.37	1.00–1.88	0.054
Current employment (*n* = 1683)	2.29	1.56–3.36	<0.001 *	1.83	1.19–2.80	0.006 *
Current health insurance coverage (*n* = 1677) ^d^	1.30	0.95–1.78	0.108	1.45	1.04–2.01	0.030 *
Loss of health insurance coverage due to the COVID-19 pandemic (*n* = 1687)	1.75	0.90–3.41	0.101	1.60	0.81–3.15	0.175
Higher education level (*n* = 1686) ^e^	2.44	1.75–3.42	<0.001 *	2.00	1.42–2.84	<0.001 *
Greater perceived accuracy of positive COVID-19 test result (*n* = 1688)	1.83	1.32–2.52	<0.001 *	1.56	1.12–2.17	0.009 *
Greater perceived accuracy of negative COVID-19 test result	1.54	0.85–2.77	0.155	1.26	0.69–2.30	0.446
High COVID-19-related worry	1.56	1.21–2.03	0.001 *	1.40	1.07–1.84	0.015 *
Receipt of ≥1 dose of a COVID-19 vaccine (*n* = 1688)	2.49	1.89–3.27	<0.001 *	2.45	1.83–3.28	<0.001 *
Belief that COVID-19 vaccination is effective	1.28	0.81–2.03	0.288	1.28	0.80–2.06	0.302

Note. aOR, adjusted odds ratio; OR, odds ratio; 95% CI, 95% confidence interval. ^a^ Multivariable models adjusted for age, sex, race/ethnicity, and income. Race/ethnicity was not included as a covariate in the model with race/ethnicity as the main predictor. Income was not included as a covariate in the model with higher income as the main predictor. ^b^ “Other race” included Asian, Native Hawaiian or Other Pacific Islander, American Indian or Alaska Native, some other reported race that was not included in the response options, or report of multiple (more than one) races. ^c^ Annual household income ≥USD 15,000 vs. <USD 15,000. “Prefer not to answer” responses were separated into a third category. ^d^ Private or public insurance (Medicare, Medicaid, Tricare) vs. no insurance/do not know. ^e^ Some college or more vs. less than high school. * *p* < 0.05.

**Table 4:** Predictors of past COVID-19 infection (self-report): Univariate and multivariable binary logistic regressions.

Predictor	OR	95% CI	*p*	aOR ^a^	95% CI	*p*
COVID-19-related challenges; challenges with:						
Obtaining necessary health care (including for mental health)	0.89	0.65–1.22	0.468	1.20	0.86–1.68	0.274
Having a place to stay/live	1.16	0.83–1.62	0.391	1.33	0.93–1.89	0.120
Sourcing enough food to eat	1.01	0.75–1.38	0.937	1.12	0.82–1.55	0.477
Having clean water to drink	1.12	0.71–1.75	0.633	1.25	0.78–1.99	0.357
Obtaining necessary medicine	1.27	0.89–1.82	0.192	1.40	0.96–2.04	0.077
Transportation	1.01	0.72–1.41	0.956	1.14	0.81–1.63	0.451
Race/Ethnicity (*n* = 1682)						
Black non-Hispanic vs. White non-Hispanic	1.10	0.53–2.27	0.795	1.14	0.55–2.35	0.733
Black Hispanic vs. White non-Hispanic	2.80	1.17–6.72	0.021 *	2.78	1.15–6.73	0.024 *
White Hispanic vs. White non-Hispanic	2.52	1.23–5.15	0.011 *	2.46	1.20–5.06	0.014 *
Other/Multiracial ^b^ vs. White non-Hispanic	2.47	1.05–5.80	0.038 *	2.40	1.02–5.68	0.046 *
Higher income (*n* = 1688) ^c^	1.71	1.30–2.25	<0.001 *	1.46	1.08–1.95	0.013 *
Current employment (*n* = 1683)	1.84	1.38–2.46	<0.001 *	1.38	0.97–1.96	0.075
Current health insurance coverage (*n* = 1677) ^d^	1.17	0.82–1.66	0.384	1.29	0.89–1.87	0.177
Loss of health insurance coverage due to the COVID-19 pandemic (*n* = 1687)	2.09	1.31–3.34	0.002 *	2.08	1.28–3.40	0.003 *
Higher education level (*n* = 1686) ^e^	1.58	1.15–2.17	0.004 *	1.32	0.94–1.84	0.110
Greater perceived accuracy of positive COVID-19 test result (*n* = 1688)	1.90	1.22–2.96	0.005 *	1.59	1.00–2.50	0.047 *
Greater perceived accuracy of negative COVID-19 test result	1.04	0.52–2.06	0.912	0.84	0.41–1.70	0.624
High COVID-19-related worry	1.30	0.98–1.72	0.070	1.08	0.81–1.46	0.597
Receipt of ≥1 dose of a COVID-19 vaccine (*n* = 1688)	1.10	0.80–1.50	0.563	0.98	0.70–1.37	0.897
Belief that COVID-19 vaccination is effective	0.80	0.50–1.28	0.350	0.78	0.48–1.28	0.327

Note. aOR, adjusted odds ratio; OR, odds ratio; 95% CI, 95% confidence interval. ^a^ Models adjusted for age, sex, race/ethnicity, income, morbidity burden, and vaccination status. Race/ethnicity was not included as a covariate in the model with race/ethnicity as the main predictor. Income was not included as a covariate for the models with higher income as the main predictor. Vaccination status was not included as a covariate for the models with belief that COVID-19 vaccination is effective as the main predictor or vaccination status as the main predictor, to avoid collinearity. ^b^ “Other race” included Asian, Native Hawaiian or Other Pacific Islander, American Indian or Alaska Native, some other reported race that was not included in the response options, or report of multiple (more than one) races. ^c^ Annual household income ≥USD 15,000 vs. <USD 15,000. “Prefer not to answer” responses were separated into a third category. ^d^ Private or public insurance (Medicare, Medicaid, Tricare) vs. no insurance/do not know. ^e^ Some college or more vs. less than high school. * *p* < 0.05.

**Table 5:** Predictors of COVID-19 positivity (rt-PCR): Univariate and multivariable binary logistic regressions ^a^.

Predictor	OR	95% CI	*p*	aOR ^b^	95% CI	*p*
COVID-19-related challenges; challenges with:						
Obtaining necessary health care (including for mental health)	1.31	0.67–2.55	0.428	1.22	0.62–2.40	0.568
Having a place to stay/live	0.97	0.45–2.09	0.930	0.77	0.34–1.71	0.518
Sourcing enough food to eat	0.85	0.42–1.72	0.647	0.71	0.35–1.47	0.359
Having clean water to drink	1.55	0.65–3.72	0.323	1.52	0.63–3.71	0.355
Obtaining necessary medicine	0.91	0.38–2.18	0.840	0.82	0.34–1.98	0.659
Transportation	1.63	0.85–3.13	0.141	1.37	0.70–2.70	0.360
Race/Ethnicity (*n* = 1682)						
Black non-Hispanic vs. White non-Hispanic	1.43	0.33–6.14	0.631	1.34	0.31–5.80	0.700
Black Hispanic vs. White non-Hispanic ^c^	-	-	-	-	-	-
White Hispanic vs. White non-Hispanic	1.21	0.27–5.36	0.800	1.16	0.26–5.22	0.845
Other/Multiracial ^d^ vs. White non-Hispanic	2.07	0.37–11.59	0.408	1.88	0.33–10.70	0.478
Higher income (*n* = 1688) ^e^	1.07	0.57–1.00	0.839	0.95	0.49–1.83	0.871
Current employment (*n* = 1683)	0.96	0.47–1.95	0.915	0.73	0.32–1.62	0.435
Current health insurance coverage (*n* = 1677) ^f^	0.85	0.42–1.72	0.647	1.02	0.48–2.16	0.955
Loss of health insurance coverage due to the COVID-19 pandemic (*n* = 1687)	0.73	0.17–3.05	0.666	0.79	0.19–3.33	0.743
Higher education level (*n* = 1686) ^g^	0.82	0.43–1.57	0.547	0.86	0.43–1.72	0.669
Greater perceived accuracy of positive COVID-19 test result (*n* = 1688)	1.20	0.50–2.85	0.685	1.37	0.56–3.31	0.490
Greater perceived accuracy of negative COVID-19 test result	0.42	0.15–1.22	0.110	0.47	0.16–1.37	0.165
High COVID-19-related worry	0.91	0.50–1.66	0.768	0.94	0.50–1.75	0.840
Receipt of ≥1 dose of a COVID-19 vaccine (*n* = 1688)	0.55	0.30–1.01	0.053	0.71	0.37–1.36	0.302
Belief that COVID-19 vaccination is effective	0.34	0.16–0.72	0.005 *	0.44	0.20–0.97	0.042 *
Past COVID-19 infection (*n* = 1688)	1.49	0.73–3.03	0.276	1.47	0.70–3.08	0.304

Note. aOR, adjusted odds ratio; OR, odds ratio; rt-PCR, real-time reverse transcription–polymerase chain reaction; 95% CI, 95% confidence interval. ^a^ We advise caution in interpreting these values as the outcome event rate was rare (2.8%), raising the possibility of a quasi-complete separation of data points. ^b^ Models adjusted for age, sex, race/ethnicity, income, morbidity burden, and vaccination status. Race/ethnicity was not included as a covariate in the model with race/ethnicity as the main predictor. Income was not included as a covariate for the models with higher income as the main predictor. Vaccination status was not included as a covariate for the models with belief that COVID-19 vaccination is effective as the main predictor or vaccination status as the main predictor, to avoid collinearity. ^c^ Unable to interpret OR due to small cell counts caused by rare outcome event rate and small proportion of those who reported Black Hispanic race/ethnicity (*n* = 47 tested positive for SARS-CoV-2 via rt-PCR testing and *n* = 73 reported Black Hispanic race/ethnicity). ^d^ ”Other race” included Asian, Native Hawaiian or Other Pacific Islander, American Indian or Alaska Native, some other reported race that was not included in the response options, or report of multiple (more than one) races. ^e^ Annual household income ≥USD 15,000 vs. <USD 15,000. “Prefer not to answer” responses were separated into a third category. ^f^ Private or public insurance (Medicare, Medicaid, Tricare) vs. no insurance/do not know. ^g^ Some college or more vs. less than high school. * *p* < 0.05.

## Data Availability

The data presented in this study are available on request from the corresponding author. The data are not publicly available due to privacy and confidentiality restrictions. Requests to access the Rapid Acceleration of Diagnostics—Underserved Populations (RADx-UP) common data elements (CDES) datasets should be directed to the RADx-UP Data Core, at radx-up-cdcc@dm.duke.edu.
